# Experimental Investigations on the Application of Natural Plant Fibers in Ultra-High-Performance Concrete

**DOI:** 10.3390/ma17143519

**Published:** 2024-07-16

**Authors:** Linus Joachim, Vincent Oettel

**Affiliations:** Institute of Concrete Construction, Leibniz University Hannover, 30167 Hannover, Germany; joachim@ifma.uni-hannover.de

**Keywords:** ultra-high-performance concrete, natural plant fibers, natural fiber reinforced concrete, post-failure behavior, performance, life cycle assessment

## Abstract

Due to its high strength, the use of ultra-high-performance concrete (UHPC) is particularly suitable for components subjected to compressive loads. Combined with its excellent durability, UHPC can be used to produce highly resource-efficient components that represent a sustainable alternative to conventional load-bearing structures. Since UHPC fails in a brittle manner without the addition of fibers, it is typically used in conjunction with micro steel fibers. The production of these steel fibers is both expensive and energy-intensive. Natural plant fibers, due to their good mechanical properties, cost-effective availability, and inherent CO_2_ neutrality, can provide a sustainable alternative to conventional steel fibers. Thanks to the low alkaline environment and dense matrix of UHPC, the use of natural plant fibers in terms of durability and bond is possible in principle. For the application of natural plant fibers in UHPC, however, knowledge of the load-bearing and post-cracking behavior or the performance of UHPC reinforced with natural plant fibers is essential. Currently, there are no tests available on the influence of different types of natural plant fibers on the load-bearing behavior of UHPC. Therefore, five series of compression and bending tensile tests were conducted. Three series were reinforced with natural plant fibers (bamboo, coir, and flax), one series without fibers, and one series with steel fibers as a reference. Under compression loads, the test specimens reinforced with natural plant fibers did not fail abruptly and exhibited a comparable post-failure behavior and damage pattern to the reference specimens reinforced with steel fibers. In contrast, the natural plant fibers did not perform as well as the steel fibers under bending tensile stress but did show a certain post-cracking bending tensile strength. A final life cycle assessment demonstrates the superiority of natural plant fibers and shows their positive impact on the environment.

## 1. Introduction

Advances in material research have made high-performance materials such as ultra-high-performance concrete (UHPC) available to the construction industry. Due to its very high compressive strengths and durability, UHPC provides a promising and forward-looking approach to resource-optimized construction, particularly for components subjected to compressive loads [1,2,3,4,5,6,7,8,9,10,11]. In order to avoid sudden and brittle failure of UHPC, UHPC is (almost) exclusively applied in combination with micro steel fibers (e.g., [1,3]). The steel fibers are added to the concrete during the manufacturing process to delay the crack formation and to sew the crack edges together [12], thus improving the post-cracking behavior of the cracked concrete (e.g., [13,14,15,16]). Depending on the steel fiber content, very high post-cracking bending tensile strengths can be achieved (post-cracking bending tensile strength > cracking bending tensile strength, e.g., [1,17,18]), which significantly positively influence the bending, shear, and torsional load-bearing behavior of UHPC components in terms of load-bearing capacity, stiffness, etc. (e.g., [19,20,21]). Based on these investigations, additive calculation approaches for bending, shear, and torsion were developed, which consider the fiber load-bearing effect (post-cracking tensile strength) in addition to the reinforcing steel when determining the load-bearing capacity (e.g., [19,22,23,24]). Thus, it is possible to consider the steel fibers not only for ductility but also statically in the design. 

However, the production of steel fibers is energy-intensive, releases comparatively high amounts of greenhouse gases, and involves high consumption of non-renewable raw materials [3]. Furthermore, the availability of steel in some regions of the world is limited, or these countries are dependent on imports with strongly fluctuating prices and must compete in the world market [25,26,27], striving to find comparable cost-effective and sustainable alternatives [28,29]. Synthetic fibers made of glass, carbon, or polymers also do not represent a real alternative for these countries, as they are similarly limited in availability and even exceed steel in cost [30,31,32,33].

Natural plant fibers, on the other hand, offer a sustainable alternative to steel fibers [34]. The use of plant-based natural fibers in polymer materials has already become established [35,36] and a study can be found in [37] that shows the advantages of natural plant fibers in polymer cementitious composites as fire protection measures. In contrast, natural plant fibers are still rarely used in concretes. The currently available studies on natural plant fiber-reinforced normal-strength concretes and mortars [38,39,40] show the potential of natural plant fibers in concrete, especially their positive effect on preventing sudden structural failure. However, these studies also revealed problems such as insufficient bonds between fibers and concrete and inadequate durability (e.g., [36,41]). These problems could potentially be largely solved by using natural plant fibers in UHPC, as UHPC’s low alkaline environment significantly improves the durability and bond properties of natural plant fibers due to its dense matrix [42]. This raises the question of whether a ductile load-bearing behavior can also be ensured in primarily compressively or flexurally stressed UHPC components through natural plant fibers, and whether the use of steel fibers can be dispensed with in these components. Previous studies on the application of individual types of natural plant fibers in UHPC have already demonstrated their potential to influence the flexural behavior [42] and to enhance the thermal-mechanical properties [43,44]. To investigate the crack-bridging effect of various natural plant fibers under compressive and bending tensile stress, as well as their impact on the residual load-bearing capacity in the post-cracking behavior of UHPC, experimental studies were conducted on cylinders and beams made of natural fiber-reinforced UHPC at the Institute of Concrete Construction (IfMa) of Leibniz University Hanover, which are reported below. This is preceded by an analysis of possible natural plant fiber types for use in UHPC as well as an analysis of studies already carried out and, finally, a life cycle assessment of the UHPC mixtures used with the various natural plant fibers.

## 2. State of Research

**Figure 1:**
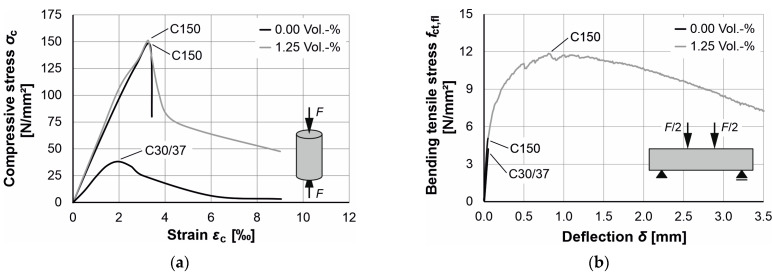
(**a**) Influence of steel fibers on the material behavior of UHPC under compressive stress; (**b**) Influence of steel fibers on the material behavior of UHPC under bending tensile stress [19].

Due to its brittle material behavior and the resulting unfavorable failure behavior, UHPC is exclusively used in combination with micro steel fibers [16,45] unless this brittleness is counteracted by strong confinement or encasement (e.g., very high stirrup reinforcement, steel tube [46]). The addition of steel fibers ensures ductile material behavior under both compressive and bending tensile stresses. Figure 1 shows the behavior of normal-strength concrete C30/37 in comparison to a UHPC without and with steel fibers under compressive stress (Figure 1a) and under flexural tensile stress (Figure 1b). Figure 1, left, clearly shows that a UHPC without steel fibers fails to brittle, whereas a UHPC with steel fibers fails to ductile and exhibits similar post-cracking behavior to a normal strength concrete C30/37. Under flexural tensile stress (Figure 1, right), the UHPC without steel fibers and the normal strength concrete C30/37 fail brittle, whereas the UHPC with steel fibers exhibits extremely ductile post-cracking behavior. 

### 2.1. Ultra-High-Performance Concrete

Ultra-high-performance concrete (UHPC) is characterized by its exceptionally high compressive strengths exceeding 130 N/mm^2^ [19,45,47]. A definition of UHPC in comparison to other concretes in terms of compressive strength, modulus of elasticity, tensile strength, rapid chloride permeability, carbonation depth, abrasion resistance, water absorption, etc. can be found in [1,48]. The remarkable load-bearing capacity is achieved through the minimization of structural flaws and the increase of packing density. This is realized through an optimized grading curve down to the fine-grain range, a significant reduction of the water-cement ratio to 0.25 to 0.15, and the use of inert and reactive additives [49]. Micro or nanosilica are often used due to their high silica content, large relative surface area, and high reactivity. The high mass proportion of pozzolans, which can be up to 30 wt.% of cement, leads to the conversion of all free calcium hydroxide, thereby lowering the alkalinity to a pH value below 10 [50]. A pH value of 10 can lead to the depassivation of the steel surface in the case of steel reinforcement [50]. However, due to the optimized microstructure and resulting high density, transport processes in UHPC are almost completely prevented, ensuring that even with minimal concrete cover, neither water nor oxygen (necessary for steel corrosion) reaches the reinforcement, thereby guaranteeing high durability [1,47,51]. Measurements of the carbonation depth of UHPC showed a carbonation progress <0.1 mm even after several years (see e.g., [1]). The reduced requirements for concrete cover and the high performance of UHPC, particularly its very high compressive strength and durability, allow for the construction of extremely thin-walled and resource-optimized components or structures that are more sustainable compared to normal-strength concretes [1,52,53,54].

However, a disadvantage of UHPC is its brittle failure. Therefore, micro steel fibers are usually added to UHPC, which significantly influences the ductility of UHPC. Due to the high proportion of pozzolans, the low water-cement ratio, and the optimized packing density, UHPC has a significantly improved contact zone compared to normal-strength concretes. This means that even with smooth, high-strength steel fibers without end anchoring or profiling and a slenderness *λ*_f_ of 60 to 100, no additional measures are required to ensure sufficient bonding of the fibers with the cement-bound matrix [55]. Additional anchoring by end hooks or profiling would rather lead to fiber breakage and thus to an undesirable brittle failure of the concrete component [56,57].

However, it should also be noted at this point that micro steel fibers have a negative impact on the sustainability of the UHPFRC [3,9,53]. This could be remedied, for example, by using natural plant fibers.

### 2.2. Natural Plant Fibers

Natural plant fibers are complex composite materials with a polylaminar cell structure. The cell walls consist of varying proportions of hemicellulose, lignin, and cellulose [58,59,60]. The cellulose molecules are arranged in chains and form the basic structure of the fibers, which has a decisive influence on the tensile properties [61]. The microfibrillar angle of inclination of the cellulose chains to the longitudinal fiber axis (MFA) also plays a significant role: with a higher MFA, both the tensile strength and the stiffness of the fibers decrease, while the strain at break increases [59,61,62].

The length of the fibers varies depending on the plant species and is usually between a few millimeters and centimeters. These fibers are present in the plants in bundles that can comprise less than 10 to over 100 individual fibers [58]. Although single fibers have a higher tensile strength [59,61], fiber bundles are mainly used in concretes and mortars. This is due to the difficult mechanical separation of the individual fibers, their small diameter of around 20 µm, and their low bending stiffness [58,59,61]. Due to the different composition, geometry, and structure of the fibers or fiber bundles, the mechanical properties of natural plant fibers vary. As can be seen in Table 1, coir has a lower tensile strength, while sisal and bamboo have a tensile strength similar to that of reinforcing steel. Flax and hemp even achieve a tensile strength comparable to that of high-strength steel.

In addition to mechanical properties, numerous other factors play a decisive role in the sustainable use of natural plant fibers in concretes worldwide. These include the use of pesticides, fungicides, and fertilizers in cultivation as well as the local availability of the fibers, particularly in so-called developing countries. Although hemp and flax fibers have advantageous mechanical properties, their cultivation is limited to temperate climate zones, which restricts their global applicability [58]. Developing countries are mainly located in tropical and subtropical regions, where coconut palms and bamboo are the most commonly used natural fiber plants [58,68,69,70]. The cultivation of coconut palms in coastal areas is particularly advantageous due to their high tolerance to salt water and their low freshwater requirement [58,71]. However, coir fibers obtained from coconuts have lower mechanical properties compared to other plant fibers (see Table 1). Bamboo, on the other hand, offers numerous advantages as a plant fiber source, including its early harvest maturity after 3 to 5 years [72,73], easy harvesting and processing, vegetative propagation by rhizomes [74], protection of the soil from erosion [75] and the positive influence on the groundwater table [25].

### 2.3. Natural Plant Fiber Reinforced Concrete

Studies on concretes reinforced with natural plant fibers in the literature focus primarily on their effect on bending tensile behavior and on concretes with lower strengths. There are already several studies on the use of bamboo fibers in concretes in particular, which demonstrate the potential of this type of fiber [76,77,78]. Detailed compilations of investigations already carried out can be found in [42,79,80]. Studies on the tensile behavior and durability of flax reinforcement are available, for example, from [81,82,83], which demonstrate the enormous potential of this reinforcement alternative. In addition, the bending tensile behavior of concrete reinforced with coir fibers was also investigated by varying the fiber content, fiber dimensions, and pre-treatment method of the natural plant fibers [36,84,85]. The tests mostly showed a fiber pull-out, which indicates a failure of the bond between the fibers and the cementitious matrix. This type of failure is aimed at in order to ensure ductile behavior [55,57]. However, this also shows that the tensile strength of the fibers is not fully utilized. Similar results were observed in other studies [86,87,88,89]. In summary, the use of fibers prevented brittle failure of the bending beams and a certain post-cracking tensile strength was determined after cracking. Comparable results were also obtained in studies on the use of natural plant fibers in sustainable landfill liners [90], asphalt mixtures [91], or double-skin columns [92].

In order to make optimum use of the tensile strength of the fibers, the slenderness of the fibers can be increased, which leads to an improved bonding effect. However, the workability of the concrete must be taken into account here, which deteriorates with increasing fiber slenderness [55,93]. It is not possible to improve the bond with plant fibers by means of end hooks or profiling [94]. Instead, the bonding of natural plant fibers can be improved by alkaline pretreatment of the fibers [41,66,95,96]. In addition, it must be noted that plant fibers absorb part of the mixing water due to their hygroscopic properties, which leads to an increase in the volume of the fibers (primary swelling) during concreting. After the concrete has hardened, however, the fibers dry, which leads to a reduction in volume and thus to a weakening of the bond between concrete and fibers [97,98,99,100]. This effect can also be caused by secondary swelling or drying out of the plant fibers in the hardened concrete due to fluctuating pore moisture [39,74,101].

In addition to the bond, the possible decomposition of the fibers in the alkaline environment of the concrete also plays a special role when using natural plant fibers in concretes. The cellulose fibers, which are decisive for tensile strength, show a certain alkali resistance compared to other fiber components. However, prolonged exposure to an alkaline environment, as is common in conventional concretes and mortars, leads to embrittlement and slow decomposition of these fibers [39,59,102], as well as a decrease in the transmissible composite voltage [41]. This can be prevented, among other things, by reducing the alkalinity of the concrete [103]. One way of limiting alkalinity is to use pozzolans. The reaction of the silicic acid contained in the pozzolans with the calcium hydroxide crystals (CH crystals), particularly in the contact zone between hydrated cement, aggregate, and fibers, leads to the formation of calcium silicate hydrate phases (CSH phases). This also contributes to improving the bond between the fibers and the cementitious matrix [47,104].

A combination of natural plant fibers with UHPC seems particularly suitable, as the low water-cement ratio of the UHPC reduces the swelling of the natural plant fibers in the concrete, the dense concrete structure increases the contact zone between fiber and concrete and improves the bond, and the limited alkalinity increases the durability.

## 3. Experimental Investigations

**Figure 2:**
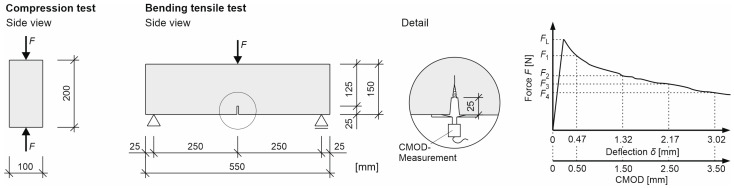
Compression test according to [106,107] (**left**) and three-point bending tensile test with typical load–deflection or load–CMOD diagram according to [105] (**right**).

In order to investigate the influence of natural plant fibers on the load-bearing and post-cracking behavior of components made of UHPC, five series of compression, stiffness, and bending tensile tests were carried out on cylinders and beams (3-point bending tensile tests analogous to [105]) made of UHPC were carried out (Figure 2). Three series with natural plant fibers, namely bamboo fibers (series 3), coir fibers (series 4), and flax fibers (series 5) were tested. The natural plant fibers were selected on the one hand to cover the widest possible range of variation in terms of tensile strength and module of elasticity (see Table 1) and thus to be able to investigate their effects on the load-bearing behavior. On the other hand, this was also to be able to investigate the variation in terms of fiber production (see Section 3.1) regarding life cycle assessment (see Section 4). In addition, a series without fibers (series 1) and a series with steel fibers (series 2) were tested as a reference. The fiber content was 1.25% by volume in each case. Each series consisted of three cylinders with a diameter of *d* = 10 cm and a height of *h* = 20 cm as well as three beams with a length of *l* = 55 cm, a width of *b* = 15 cm, and a height of *h* = 15 cm. The compressive strengths and stiffnesses (compressive modulus of elasticity) were determined in a compression testing machine in accordance with [106,107], and the bending tensile strengths were determined in a universal testing machine according to [105] (see also [18,108]). The test program is summarized in Table 2.

### 3.1. Test Specimens

The test specimens were produced in cylindrical formwork or in beam formwork with ultra-high-performance concrete. The UHPC composition can be seen in Table 3 and was based on the concrete compositions with micro steel fibers of SPP 1182 “Building Sustainable with Ultra-High-Performance Concrete (UHPC)” [109] and the SPP 2020 “Cyclic deterioration of High-Performance Concrete in an experimental-virtual lab” [110] of the German Research Foundation (DFG) (see also [11,13,16,19,45]). For all mixtures, the w/c ratio was 0.19 and the superplasticizer content was 4.54 wt% relative to the cement.

The characteristic values of the fibers regarding their geometry and raw density can be found in Table 4 and the chemical composition of the natural plant fibers used can be found in Table 5.

**Figure 3:**
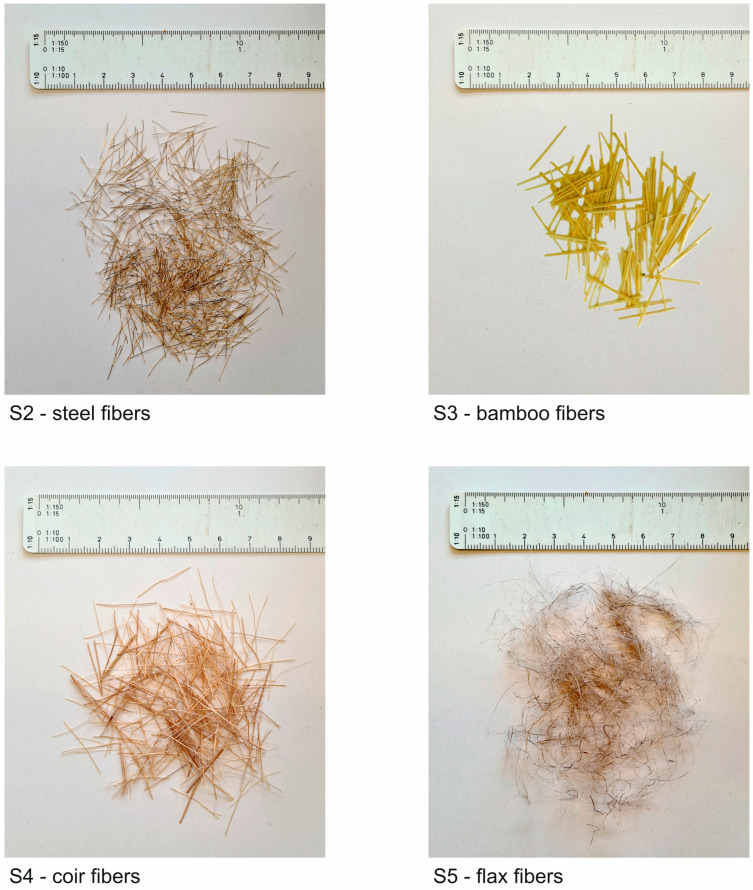
Fibers used in the S2 to S5 series (unit of the ruler in cm).

The steel fibers were smooth, straight micro steel fibers with a tensile strength of >2000 N/mm^2^ (Figure 3, top left). The natural plant fibers were produced by hand. The production of the bamboo fibers used in the tests (Figure 3, top right) involved the most effort compared to the production of the other natural plant fibers. The bamboo fibers were obtained mechanically from the culm wall of a moso bamboo (Latin name: Arundinarieae Arundinariinae Phyllostachys Pubescens). A detailed description of the production process can be found in [42].

The production of the coir fibers required the least effort, as they were simply cut to a length of 15 to 20 mm using a cutting machine. The diameter of 0.2 to 0.8 mm was already predetermined by the raw material (Figure 3, bottom left). Compared to the production of coir fibers, the production of flax fibers involved a bit more effort. The original state for the production of flax fibers was 90 cm long flax fiber bundles. First, they were combed to separate individual fibers from the bundles. Afterward, the fibers were cut into a length of 15 to 20 mm using a cutting machine. The diameter here was also specified by the raw material at ≤0.2 mm (Figure 3, bottom right).

After concreting the test specimens, they were covered with foil, stripped of their formwork the following day, and stored at room temperature until the test was carried out. Before the test was carried out, the cylinders were ground flat and the beams were notched with a concrete saw about 2.5 cm deep in the middle, with a notch width of about 0.5 cm, so that an effective height of about 12.5 cm was available (Figure 2, see also [105]).

### 3.2. Test Execution

As already mentioned, the compressive strength and modulus of elasticity were determined in a compression testing machine, and the bending tensile strength in a universal testing machine. The three-point bending tensile test was carried out in accordance with EN 14651 [105] (Figure 2 right, see also [18,108]). In addition to the vertical test force *F*, the relative vertical displacement was measured and documented in the cylinder tests with three lasers and in the bending tensile tests with two inductive displacement transducers. The cylinder tests were force-controlled at 0.5 MPa/s and the bending tensile tests were displacement-controlled at 0.1 mm/min. The bending tensile tests were terminated after reaching a vertical feed path of approx. 3.5 mm [18]. In order to obtain as much information as possible on the failure of the cylinder specimens, the compression tests were recorded with a high-speed camera at a frame rate of 8000 frames per second. A subsequent slow-motion analysis made it possible to precisely detect the formation and growth of cracks as well as the failure of the test specimens.

### 3.3. Test Results and Discussions

#### 3.3.1. Compression Tests

**Figure 4:**
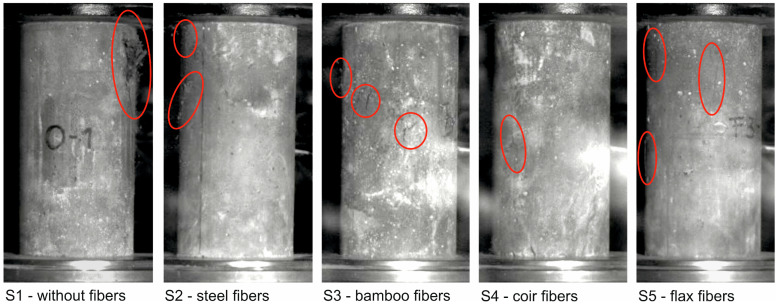
UHPC cylinder at the time of initial cracking (images from a high-speed camera).

**Figure 5:**
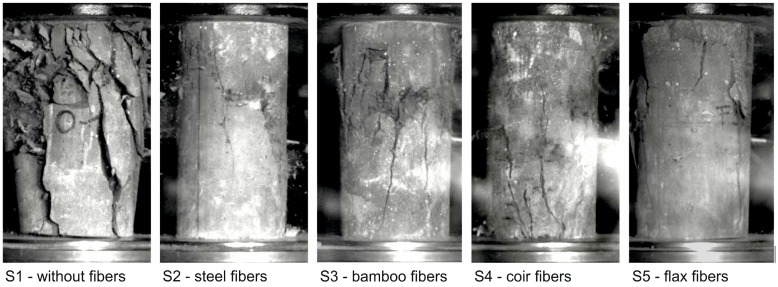
UHPC cylinder at the time of failure (images from a high-speed camera).

**Figure 6:**
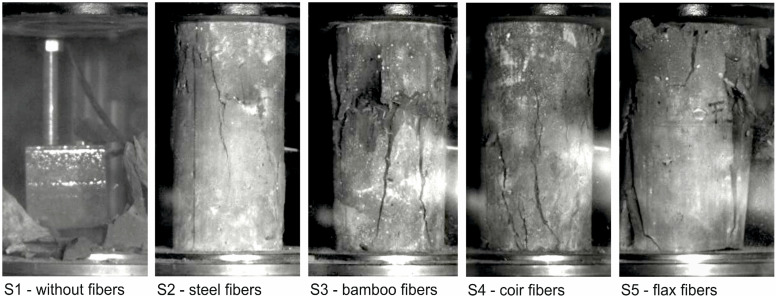
UHPC cylinder after the test (images from a high-speed camera).

When testing the cylinders without fiber reinforcement, a sudden and brittle failure occurred as expected, while in all cylinder tests with fiber reinforcement—both with steel and natural plant fibers—ductile rather than sudden failure occurred. The photos of the test specimens at the time of initial cracking are shown in Figure 4 (cracks are marked with a red circle), at the time of failure are shown in Figure 5, and after the test in Figure 6 (images from a high-speed camera).

**Figure 7:**
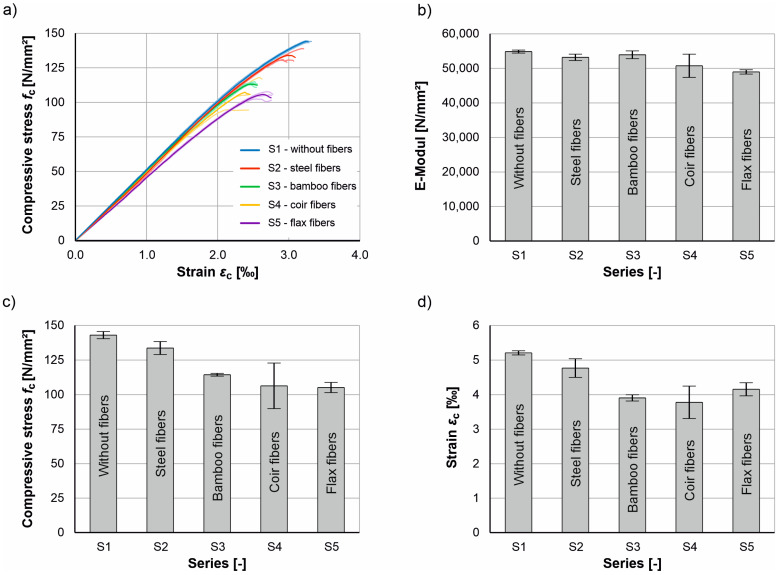
Results of the compression tests: (**a**) stress-strain diagram and comparison of the (**b**) modulus of elasticity, (**c**) compressive stresses, and (**d**) strains at failure of the series S1 to S5.

The images from the high-speed camera clearly show that the unreinforced UHPC fails explosively under axial compressive load (Figure 5, left) and then breaks up into several fragments (Figure 6, left). In contrast, only marginal differences can be seen in the crack patterns of the fiber-reinforced UHPC cylinders at the time of failure (Figure 5) compared to the completed crack patterns at the end of the test (Figure 6). While minor subsequent spalling can still be seen in the cylinders reinforced with bamboo and flax fibers, no further changes occurred in the crack patterns of the cylinders reinforced with steel and coir fibers. The individual lines and the respective mean value lines of the stress-strain relationships as well as the mean value of the modulus of elasticity, the maximum compressive stress, and the corresponding strain at failure including scattering of the tested series S1 to S5 are shown in Figure 7. As the cylinders could only be tested under load control (see Section 3.2), the stress-strain relationships in Figure 7a are only shown up to the maximum load (in contrast to Figure 1a).

It can be seen that the addition of fibers has a negative effect on the stiffness of the UHPC (Figure 7b), with the reduction depending on the fiber type ranging from very low ≈ 2–3% (for steel and bamboo fibers) to approx. 10% (for coir and flax fibers). In contrast, the compressive stresses (Figure 7c) and the associated strains (Figure 7d) show greater differences between the unreinforced and fiber-reinforced cylinders. While the cylinders reinforced with steel fibers show reductions of 6.6% in the maximum compressive stresses and 8.4% in the associated strains, these are significantly higher for the cylinders reinforced with natural plant fibers, namely between 20% and 26.4% in the compressive stresses and between 20.2% and 27.4% in the associated strains. 

The decrease in compressive strength and modulus of elasticity in UHPCs with fibers compared to UHPC without fibers can be explained by air voids. The addition of the fibers to the fresh concrete introduces air into the fresh concrete and makes it more difficult to work, resulting in an increased proportion of compaction voids. In the case of UHPCs with natural plant fibers, a further reason could be that the natural plant fibers remove part of the mixing water during the mixing process so that the actual w/c ratio is lower and less cement can hydrate. Apart from this reason, natural plant fibers may lead to more serious degradation of the interfacial transition zone (ITZ) between the cement paste and aggregate, which also results in a decrease in compressive strength. For the tests presented here, a UHPC composition developed for micro steel fibers was used (see Section 3.1). By adapting the mix, e.g., by adding more water and/or superplasticizer, the reduction in compressive strength when using natural plant fibers could probably be completely eliminated. 

Figure 7 also shows that the scatter of stiffnesses, compressive stresses, and associated strains are comparable for the series tested, with the exception of series 4 “Coir fibers”. A compilation of the characteristic values (mean values) of the compression tests and their standard deviation ($s_{f_{c}}$, $s_{\epsilon_{c}}$ and $s_{E}$) is listed in Table 6.

#### 3.3.2. Bending Tensile Tests

**Figure 8:**
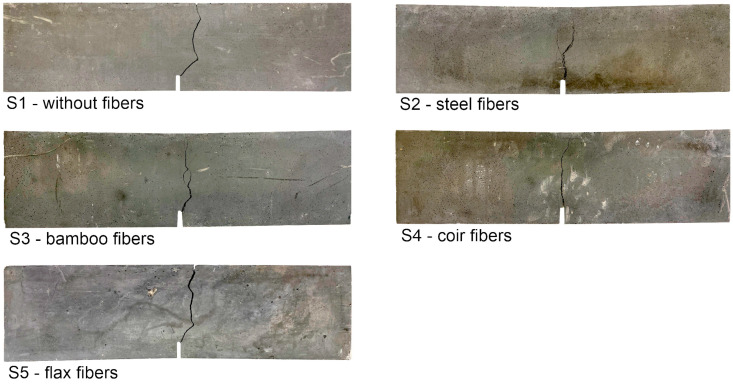
Bending beams after the test (side view).

**Figure 9:**
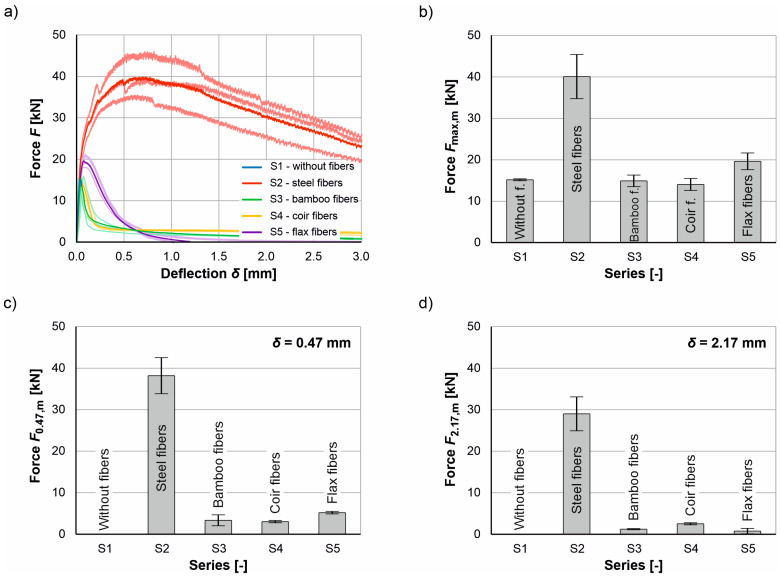
Results of the bending tensile tests: (**a**) force-deflection diagram and comparison of the (**b**) maximum loads, (**c**) loads at a deflection of 0.47 mm (CMOD_1_), and (**d**) loads at a deflection of 2.17 mm (CMOD_3_) of series S1 to S5.

As expected, testing of the bending beams without fiber reinforcement also resulted in sudden and brittle failure. In contrast, ductile material behavior was observed in the series with fiber reinforcement. Figure 8 shows the test specimens after the test. Figure 9 shows the individual lines and the corresponding mean value lines of the force-deflection curves as well as the mean value of the maximum load *F*_max,m_, the load *F*_0.47,m_ at a deflection of 0.47 mm (=CMOD_1_ = 0.5 mm) and the load *F*_2.17,m_ at a deflection of 2.17 mm (=CMOD_3_ = 2.5 mm) of the tested series S1 to S5. The bearing loads *F*_0.47,m_ and *F*_2.17,m_ were calculated analogously [105,113], as these are to be used for the design of steel fiber reinforced concrete (see [18,24,108,114]).

The force-deflection curves are linear for all series up to the initial crack formation. However, cracking leads to a loss of stiffness, and the test load drops. In the case of the bending beams reinforced with steel fibers, the force then increases again and reaches its maximum load at an average deflection of between 0.5 mm and 0.7 mm. The test load then decreases steadily with increasing deflection (see also [18]). The initial cracking loads of the bending beams reinforced with natural plant fibers are significantly lower than those of the bending beams reinforced with steel fibers at a deflection < 0.1 mm (≈initial cracking). Depending on the natural fiber reinforcement, there are minor differences in the maximum loads (see Figure 9b). In contrast to the bending beams reinforced with steel fibers, the load-bearing capacity of the bending beams reinforced with natural plant fibers decreases with increasing deflection after initial cracking. Both the coir and the bamboo fibers were able to ensure a certain residual load-bearing capacity due to their crack-bridging effect and were slowly pulled out of the concrete matrix as the test progressed. This is an intended failure mode for UHPC under tensile loading (e.g., [13,14,16,24,108]). The bending beams reinforced with flax fibers, on the other hand, were able to withstand slightly higher loads but broke at the end of the test (see Figure 8, bottom left). These results show that the tensile strength of the natural plant fibers (see Table 1) does not seem to have any influence on the behavior after cracking. Furthermore, it can be seen that the bending tensile strength of the beams reinforced with natural plant fibers is only 10% on average at a deflection of 0.47 mm and only 5% on average at a deflection of 2.17 mm compared to the beams reinforced with steel fibers. Overall, the results of the bending tensile tests show that for bending tensile-stressed UHPC beams reinforced with natural plant fibers, a sudden and brittle failure can be prevented, but the crack-bridging effect of these fibers is not significant and therefore the post-cracking bending tensile strength is significantly lower than that of UHPC beams reinforced with steel fibers. A summary of the characteristic values (mean values) of the bending tensile tests and their standard deviation ($s_{F_{max,m}}$, $s_{F_{0.47,m}}$ and $s_{F_{2.17,m}}$) is given in Table 7.

In the analysis, it should be noted that the UHPC mixture used was developed for steel fibers (see Section 3.1) and in this study for the series S3, S4, and S5 only the proportion of steel fibers was replaced by natural plant fibers, but the composition was not changed. As already mentioned in the compression tests in Section 3.2, the natural plant fibers remove water from the concrete during the mixing process, so that the actual w/c ratio is lower. As a result, less cement hydrates, creating a weaker transition zone (ITZ) between the hardened cement paste and the fibers. This leads to a weaker bond between concrete and fibers and thus to a lower load-bearing capacity under bending tensile stress. This phenomenon can probably be counteracted by concrete technology measures such as improving the concrete mixture by increasing the water and/or superplasticizer content.

## 4. Life Cycle Assessment

Investigations on the sustainability of components made of UHPC, e.g., according to [1,3,9,53], show that at the material level, in addition to the very high cement content, the micro steel fibers in particular have very high GWP values and have a negative impact on the life cycle assessment. With regard to the reduction of the cement content through the use of secondary cementitious materials (fly ash, ground granulated blast-furnace slag, etc.), initial studies have been carried out according to [115,116,117]. Further investigations with regard to the development of green UHPC, e.g., [118,119,120] deal with the replacement of fine aggregates with construction and demolition waste. With regard to micro steel fibers, initial investigations using recycled micro steel fibers in [120,121,122] are available. In contrast, to the authors’ knowledge, there are currently no studies on the life cycle assessment of UHPC with natural plant fibers. In order to investigate the influence of natural plant fibers on the LCA of UHPC, the UHPC mixture used here was therefore ecobalanced with the different fiber types (see Table 3).

As part of a life cycle assessment of building materials or structures, the input and output flows of the potential environmental impacts of a system are summarized over the entire life cycle, and the influencing variables are evaluated [123]. The entire life cycle of a building is divided into so-called life cycle phases (see [124]). The first phase A1 to A3 relates to the product, the second phase A4 to A5 to the construction process, the third phase B1 to B7 to the use, and the fourth phase C1 to C4 to the end of life. In addition, there is a fifth phase D, which deals with the benefits and loads beyond the system boundary. In EN 15804 [125], indicators such as the Global Warming Potential (GWP), the Acidification Potential (AP), the Eutrophication Potential (EP), or the Ozone Depletion Potential (ODP) are provided to assess the potential environmental impacts. In the context of life cycle assessment in the construction industry, however, only the GWP (i.e., the quantities of greenhouse gas emissions) has so far been used as an indicator. 

### 4.1. Assessment

This article focuses on the calculation of the global warming potential (GWP) of the first life cycle phase A1 to A3 (A1: Raw Material Supply; A2: Transport; A3: Manufacturing) for the individual UHPC mixtures under consideration. Table 8 provides an overview of the GWP impact indicators including the data source of the components used for the life cycle phase A1 to A3. Table 8 shows that steel fibers and superplasticizers have the highest GWP values and the GWP value of the cement considered here is about half as high. It also shows that bamboo, coir, and flax fibers have approximately the same GWP values of around 0.40 kg CO_2_/kg and only achieve around 15 to 21% of the GWP value of steel fibers.

**Figure 10:**
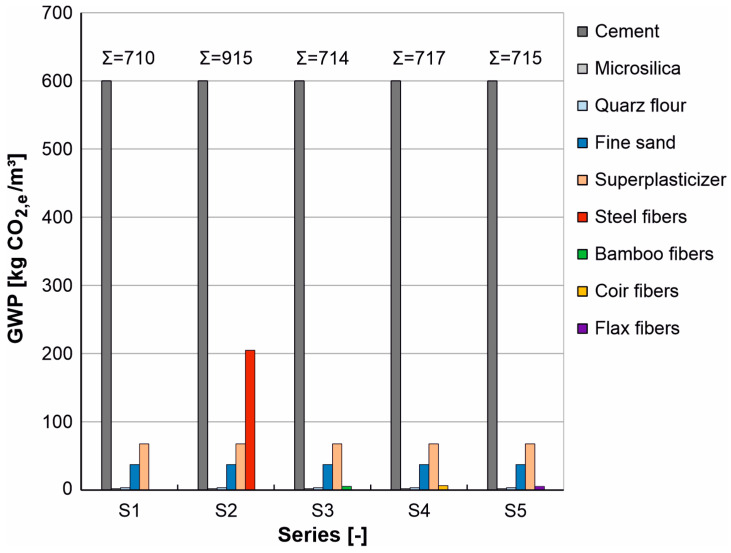
Global Warming Potential (GWP) for the investigated UHPC compositions (series S1 to S5).

For the life cycle assessment of the individual UHPC mixtures based on the global warming potential (GWP), the quantities of the individual materials of the respective mixture (see Table 3) were multiplied by the corresponding impact indicators (see Table 8). Figure 10 shows the resulting global warming potential (GWP) of the individual components for each mixture in order to enable an assessment of their contribution to the respective total GWP for the life cycle phase A1 to A3 under consideration.

### 4.2. Discussion

The evaluation shows that the GWP of the cement accounts for the largest proportion of all UHPC mixtures considered (Figure 10, gray bars). Since the UHPC mixtures considered here differ only in the fibers (see Table 3), this proportion is identical for S1 to S5 (this also applies to the other components, with the exception of the fibers). Corresponding measures to reduce the GWP share of the cement, e.g., by using secondary cement substitutes, were not considered further in this article. The steel fibers have the second largest GWP share (see Figure 10, red bar at S2), which, even with a comparatively low fiber content of 1.25% by volume, have a considerable share in the CO_2_ balance and account for around 22% of the total GWP of the S2 series. This confirms the above-mentioned investigations according to [1,3,9,53]. In addition to the cement, the micro-steel fibers in particular have very high GWP values and have a negative impact on the life cycle assessment. In comparison, the GWP share of the bamboo fibers (Figure 10, green bar at S3), the coir fibers (Figure 10, yellow bar at S4), and the flax fibers (Figure 10, purple bar at S5) is only about 1% of the respective total GWP of the series S3, S4 and S5, so that the total GWP of the series with natural plant fibers (S3, S4 and S5) hardly differs from the total GWP of the UHPC without fibers. In relation to the GWP share of the steel fibers, the GWP share of the natural plant fibers is only around 2 to 3%. 

From all these results, it can be concluded for life cycle phases A1 to A3 that natural plant fibers significantly improve the sustainability of UHPC and have great potential.

## 5. Conclusions and Outlook

Due to its very high strength and very high durability, UHPC can be a sensible and resource-efficient alternative to normal or high-strength concretes, depending on the field of application. However, as UHPC fails to brittle without the addition of fibers, micro steel fibers are usually added to UHPC, which in turn has an extremely negative impact on its ecological balance. A sustainable alternative to steel fibers is natural plant fibers. Natural plant fibers have very good mechanical properties, are cost-effective, and in principle CO_2_-neutral. The advantages of using natural plant fibers in UHPC are that the low w/c ratio limits possible swelling of the fibers, the dense structure ensures a sufficient bond and the very low alkaline environment ensures the durability of the natural plant fibers. However, there is hardly any knowledge about the load-bearing behavior of UHPC reinforced with natural plant fibers. In order to investigate the potential of natural plant fibers in UHPC, this study analyzed possible natural plant fibers for use in UHPC and, on this basis, tested the use of three selected natural fiber types, namely fibers out of bamboo, coir, and flax in UHPC under compressive and tensile bending loads and compared two reference series (without fibers and with steel fibers). In addition, a life cycle assessment of the tested UHPC with the different fiber types was carried out. The investigations yielded the following results:

- Natural plant fibers are a sustainable alternative to conventional steel fibers in terms of their worldwide distribution and cultivation possibilities, rapid growth, easy harvesting and processing, and excellent mechanical properties;
- The modulus of elasticity of the UHPC is only slightly influenced by the addition of natural plant fibers—similar to the addition of steel fibers;
- Under axial compressive loading, the addition of natural plant fibers in the post-cracking area can prevent brittle failure of the UHPC by bridging the crack edges. However, the maximum compressive stresses and the associated strains of the test specimens reinforced with natural plant fibers are reduced by between 14 and 21% compared to the reference test specimens reinforced with steel fibers;
- Under bending tensile stress, a certain residual load-bearing capacity can be ensured for the test specimens reinforced with natural plant fibers due to the crack-bridging effect, but after cracking and the associated loss of stiffness—in contrast to the bending beams reinforced with steel fibers—no renewed increase in the test load can be recorded. In addition, the initial crack loads are significantly lower than those of the bending beams reinforced with steel fibers. The post-cracking bending tensile strength for the maximum value *F*_max,m_ is only around 35 to 49% on average compared to micro steel fibers;
- The use of natural plant fibers instead of steel fibers leads to a significant reduction in global warming potential (GWP), as the GWP share can be reduced from around 22% to around 1%.

In the tests carried out, only untreated natural plant fibers and one UHPC formulation with one fiber content and one fiber slenderness per series were tested. For a better understanding of the application possibilities of natural plant fibers in UHPC, these parameters should be varied and examined more closely in further investigations, as the experimental investigations carried out clearly show the potential of natural plant fibers in UHPC to avoid sudden and brittle failure as well as a clear superiority in terms of life cycle assessment. The combination of natural plant fibers and UHPC can be a promising construction method, especially with regard to thin-walled and material-optimized components. For primarily compression-stressed components made of UHPC or compression zones in a bending-stressed cross-section, a sudden, brittle failure can be prevented using sustainable and cost-effective natural plant fibers, whereby the reduction in compressive strength would have to be taken into account based on the results to date.

This reduction could possibly be reduced by adjustments on the concrete technology side by preventing or taking into account that natural plant fibers remove water from the concrete during the mixing process and thus less cement gets hydrated. The use of superplasticizers, for example, could address this problem and may also lead to mitigation in the previously observed strength reduction, which could be associated with the increased number of micro damages inside the concrete. Although natural plant fibers can prevent brittle failure in UHPC components subjected to bending stress, the post-cracking tensile strength is significantly lower than that of UHPC reinforced with steel fibers, which means that the use of natural plant fibers in components subjected to bending stress or the use of post-cracking tensile strength in the design can currently only be classified as suitable to a limited extent. By adapting the UHPC mixture and appropriate fiber pre-treatment, the post-cracking tensile strength could possibly be increased and the potential for use as minimum reinforcement could exist.

As research into the application of natural plant fibers in UHPC is still in its infancy, there are currently no material models available for numerical simulation. However, it is very likely that the material models for UHPC with steel fibers (e.g., according to [17,18]) can be used as a basis and modified accordingly. This will require appropriate investigations.

## Figures and Tables

**Table 1:** Properties of various selected natural plant fiber bundles [58,63,64,65,66,67].

Fiber	Cellulose [%]	MFA [°]	Tensile Strength [N/mm^2^]	Module of Elasticity [N/mm^2^]
Bamboo	60	8	400	35,000
Coir	35	45	150	5000
Flax	70	7	1000	60,000
Sisal	65	17	450	40,000
Hemp	65	4	700	50,000

**Table 2:** Experimental program.

Series	Fiber Type [-]	Fiber Content [Vol.-%]	Number of Cylinders [-]	Cylinder Geometry [cm]	Number of Beams [-]	Beam Geometry [cm]
S1	-	0	3	*d*/*h* = 10/20	3	*l*/*b*/*h* = 55/15/15
S2	Steel fibers	1.25	3	*d*/*h* = 10/20	3	*l*/*b*/*h* = 55/15/15
S3	Bamboo fibers	1.25	3	*d*/*h* = 10/20	3	*l*/*b*/*h* = 55/15/15
S4	Coir fibers	1.25	3	*d*/*h* = 10/20	3	*l*/*b*/*h* = 55/15/15
S5	Flax fibers	1.25	3	*d*/*h* = 10/20	3	*l*/*b*/*h* = 55/15/15

**Table 3:** UHPC composition (kg/m^3^).

Components of the Concrete	S1	S2	S3	S4	S5
CEM I 52.5 R-SR3/NA (Holcim Sulfo 5R, Sehnde, Germany)	795	795	795	795	795
Silica fume (Sika^®^ Silicoll P (uncompacted), Stuttgart, Germany)	169	169	169	169	169
Quartz flour (Quarzwerke MILLSIL^®^ W12, Frechen, Germany)	198	198	198	198	198
Fine sand (0/0.5 mm) (Quarzwerke H33)	971	971	971	971	971
Superplasticiser (BASF MasterGlenium^®^ ACE 394, Staßfurt, Germany)	36	36	36	36	36
Steel fibers (Stratec Weidacon FM 0.19 × 13 mm, Hemer, Germany)	-	98	-	-	-
Bamboo fibers	-	-	10	-	-
Coir fibers	-	-	-	15	-
Flax fibers	-	-	-	-	18
Adding water	188	188	188	188	188

**Table 4:** Characteristic values of the fibers.

Fiber	Length [mm]	Diameter [mm]	Raw Density [kg/m^3^]
Steel	13	0.19	7850
Bamboo	20	0.5–1.0	800
Coir	15–20	0.2–0.8	1200
Flax	15–20	≤0.2	1450

**Table 5:** Chemical composition of the tested natural plant fibers [65,111,112].

Fiber	Pectin [wt%]	Hemicellulose [wt%]	Lignin [wt%]	Cellulose [wt%]
Bamboo	0.5–1.5	20–25	10–30	45–65
Coir	1–3	<1	40–45	30–40
Flax	1–4	11–18	2–3	60–75

**Table 6:** Characteristic values from the compression tests.

Series	Fiber Type [-]	$f_{c}$ [N/mm^2^]	$s_{f_{c}}$ [N/mm^2^]	$\epsilon_{c}$ [‰]	$s_{\epsilon_{c}}$ [‰]	E-Modul [N/mm^2^]	$s_{E}$ [N/mm^2^]
S1	-	143.0	2.6	3.25	0.06	54,867	439
S2	Steel fibers	133.6	4.7	2.98	0.27	53,185	926
S3	Bamboo fibers	114.4	1.0	2.44	0.09	53,948	1126
S4	Coir fibers	106.4	16.5	2.36	0.47	50,742	3364
S5	Flax fibers	105.2	3.7	2.60	0.19	48,981	622

**Table 7:** Characteristic values from the bending tensile tests.

Series	Fiber Type [-]	$F_{max,m}$ [kN]	$s_{F_{max,m}}$ [kN]	$F_{0.47,m}$ [kN]	$s_{F_{0.47,m}}$ [kN]	$F_{2.17,m}$ [kN]	$s_{F_{2.17,m}}$ [kN]
S1	-	15.16	0.18	0.00	0.00	0.00	0.00
S2	Steel fibers	40.22	5.33	38.20	4.36	29.00	4.10
S3	Bamboo fibers	14.90	1.40	3.35	1.31	1.23	0.14
S4	Coir fibers	14.02	1.45	3.03	0.27	2.52	0.25
S5	Flax fibers	19.61	2.02	5.19	0.30	0.77	0.84

**Table 8:** Impact indicators of the individual components for life cycle phase A1 to A3.

Components of the Concrete	GWP [kg CO_2,e_/kg] A1–A3	Data Source
Cement CEM I 52.5R	0.755	[126]
Microsilica	0.0061	[127]
Quartz flour	0.0181	[128]
Fine sand	0.0385	[127]
Superplasticizer	1.88	[129]
Steel fibers	2.09	[127]
Bamboo fibers	0.38	[130]
Coir fibers	0.45	[131]
Flax fibers	0.31	[127]

## Data Availability

The original contributions presented in the study are included in the article, further inquiries can be directed to the corresponding author.
